# Breathomics: may it become an affordable, new tool for early diagnosis of non-small-cell lung cancer? An exploratory study on a cohort of 60 patients

**DOI:** 10.1093/icvts/ivae149

**Published:** 2024-09-03

**Authors:** Debora Brascia, Giulia De Iaco, Teodora Panza, Francesca Signore, Graziana Carleo, Wenzhe Zang, Ruchi Sharma, Pamela Riahi, Jared Scott, Xudong Fan, Giuseppe Marulli

**Affiliations:** Department of Biomedical Sciences, Humanitas University, Pieve Emanuele, Milan, Italy; Division of Thoracic Surgery, IRCCS Humanitas Research Hospital, Rozzano, Milan, Italy; Thoracic Surgery Unit, Department of Precision and Regenerative Medicine and Jonic Area, University Hospital of Bari, Bari, Italy; Thoracic Surgery Unit, Department of Precision and Regenerative Medicine and Jonic Area, University Hospital of Bari, Bari, Italy; Thoracic Surgery Unit, Department of Precision and Regenerative Medicine and Jonic Area, University Hospital of Bari, Bari, Italy; Thoracic Surgery Unit, Department of Precision and Regenerative Medicine and Jonic Area, University Hospital of Bari, Bari, Italy; Department of Biomedical Engineering, University of Michigan, Ann Arbor, MI, USA; Department of Biomedical Engineering, University of Michigan, Ann Arbor, MI, USA; Department of Biomedical Engineering, University of Michigan, Ann Arbor, MI, USA; Department of Biomedical Engineering, University of Michigan, Ann Arbor, MI, USA; Department of Biomedical Engineering, University of Michigan, Ann Arbor, MI, USA; Department of Biomedical Sciences, Humanitas University, Pieve Emanuele, Milan, Italy; Division of Thoracic Surgery, IRCCS Humanitas Research Hospital, Rozzano, Milan, Italy

**Keywords:** Breathomic, Non-small-cell lung cancer, Screening, Volatile organic compounds, Gas chromatography

## Abstract

**OBJECTIVES:**

Analysis of breath, specifically the patterns of volatile organic compounds (VOCs), has shown the potential to distinguish between patients with lung cancer (LC) and healthy individuals (HC). However, the current technology relies on complex, expensive and low throughput analytical platforms, which provide an offline response, making it unsuitable for mass screening. A new portable device has been developed to enable fast and on-site LC diagnosis, and its reliability is being tested.

**METHODS:**

Breath samples were collected from patients with histologically proven non-small-cell lung cancer (NSCLC) and healthy controls using Tedlar bags and a Nafion filter attached to a one-way mouthpiece. These samples were then analysed using an automated micro portable gas chromatography device that was developed in-house. The device consisted of a thermal desorption tube, thermal injector, separation column, photoionization detector, as well as other accessories such as pumps, valves and a helium cartridge. The resulting chromatograms were analysed using both chemometrics and machine learning techniques.

**RESULTS:**

Thirty NSCLC patients and 30 HC entered the study. After a training set (20 NSCLC and 20 HC) and a testing set (10 NSCLC and 10 HC), an overall specificity of 83.3%, a sensitivity of 86.7% and an accuracy of 85.0% to identify NSCLC patients were found based on 3 VOCs.

**CONCLUSIONS:**

These results are a significant step towards creating a low-cost, user-friendly and accessible tool for rapid on-site LC screening.

**CLINICAL REGISTRATION NUMBER:**

ClinicalTrials.gov Identifier: NCT06034730.

## Graphical Abstract

**Figure:**
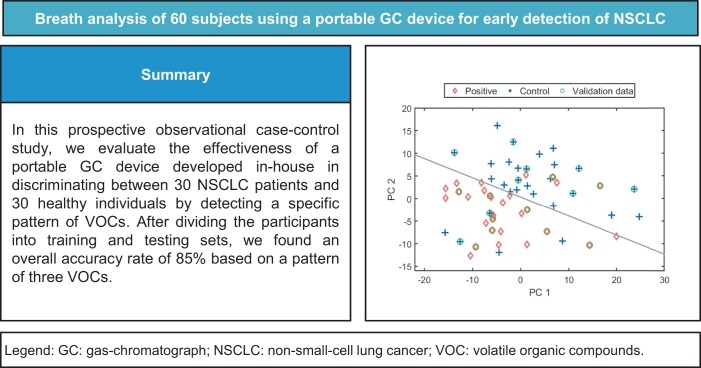

## INTRODUCTION

Lung cancer (LC) is the leading cause of cancer deaths in both men and women worldwide. Typically, LCs are asymptomatic until later stages, leading to delayed diagnosis and treatment. However, patients with stage I LC have a survival rate of >60% after 5 years [1], and those detected through computed tomography (CT) screening can have an estimated 10-year survival rate of 88% [2]. Early detection is therefore crucial to improve survival rates. Unfortunately, current methods for early detection of LC are not very reliable. Non-invasive tests like sputum cytology have low sensitivity, chest X-rays are radioactive and have a high rate of false-negative results, and low-dose CT scans are recommended for high-risk individuals but have disadvantages like a high false-positive rate, overdiagnosis and high cost, limiting their application in population-based screening [3]. Alternatively, more accurate approaches for LC diagnosis such as bronchoscopy, needle or surgical biopsy are very invasive, costly and time-consuming [4]. Metabolomics is a cutting-edge branch of ‘omics’ research that employs advanced high-throughput techniques like gas chromatography coupled to mass spectrometry (GCMS), nuclear magnetic resonance spectroscopy and high-performance liquid chromatography to investigate the end products of cellular metabolism [5]. Scientists study the endogenous volatiles produced by the body’s biochemical processes, as many diseases are thought to have distinct metabolomic profiles. Exhaled breath contains several classes of volatile organic compounds (VOCs) that could potentially serve as biomarkers for LC. Breath analysis is a promising medical diagnostic tool due to its non-invasiveness, affordability, and ease for patients to comply with [6]. However, previous studies on LC have been limited by small sample sizes, collection methods, different analytical approaches, and data processing techniques and there is currently no consensus on VOC biomarkers for LC.

This exploratory study aims to investigate whether patients with LC have a specific pattern of VOCs compared with healthy controls, by using a portable gas chromatography (GC) device developed in-house.

## MATERIALS AND METHODS

### Ethical statement

This study was approved by the Ethics Committee of the University Hospital of Bari on the 22nd of November 2021, with ID number 665139. The research was conducted in accordance with the principles embodied in the Declaration of Helsinki and in accordance with the local statutory requirements. Written informed consent for the storage and sharing of data was collected before breath-testing from either patients or non-cancer controls recruited.

This prospective observational case–control study was designed in 2 phases. The aim of the initial trial phase was to identify and select VOCs of interest, and to set up a VOC pattern potentially capable of discriminating between patients with non-small-cell lung cancer (NSCLC) and normal controls using an appropriate statistical model. The aim of the subsequent validation phase was prospectively to validate the model in a blinded fashion on a further series of patients and healthy controls; these subjects were not included in the previous phase.

### Patient population

Between August 2021 and March 2022, a total of 60 patients who were diagnosed with NSCLC and a comparative group of non-cancer controls, who had negative findings on preoperative chest X-rays/chest CT scans, were admitted to our Thoracic Surgery Unit. Before surgery, breath samples were collected during the pre-hospitalization analysis from the NSCLC patients. The control group was composed of patients who underwent surgery for benign extra-thoracic disease, had undergone chest X-rays/chest CT scans and were found to be negative during preoperative evaluation. Patients who had any history of another type of cancer and those who had received neoadjuvant chemo/radiotherapy were excluded because of the possible unknown effects on cancer metabolism.

### Description of the portable GC device

**Figure 1:**
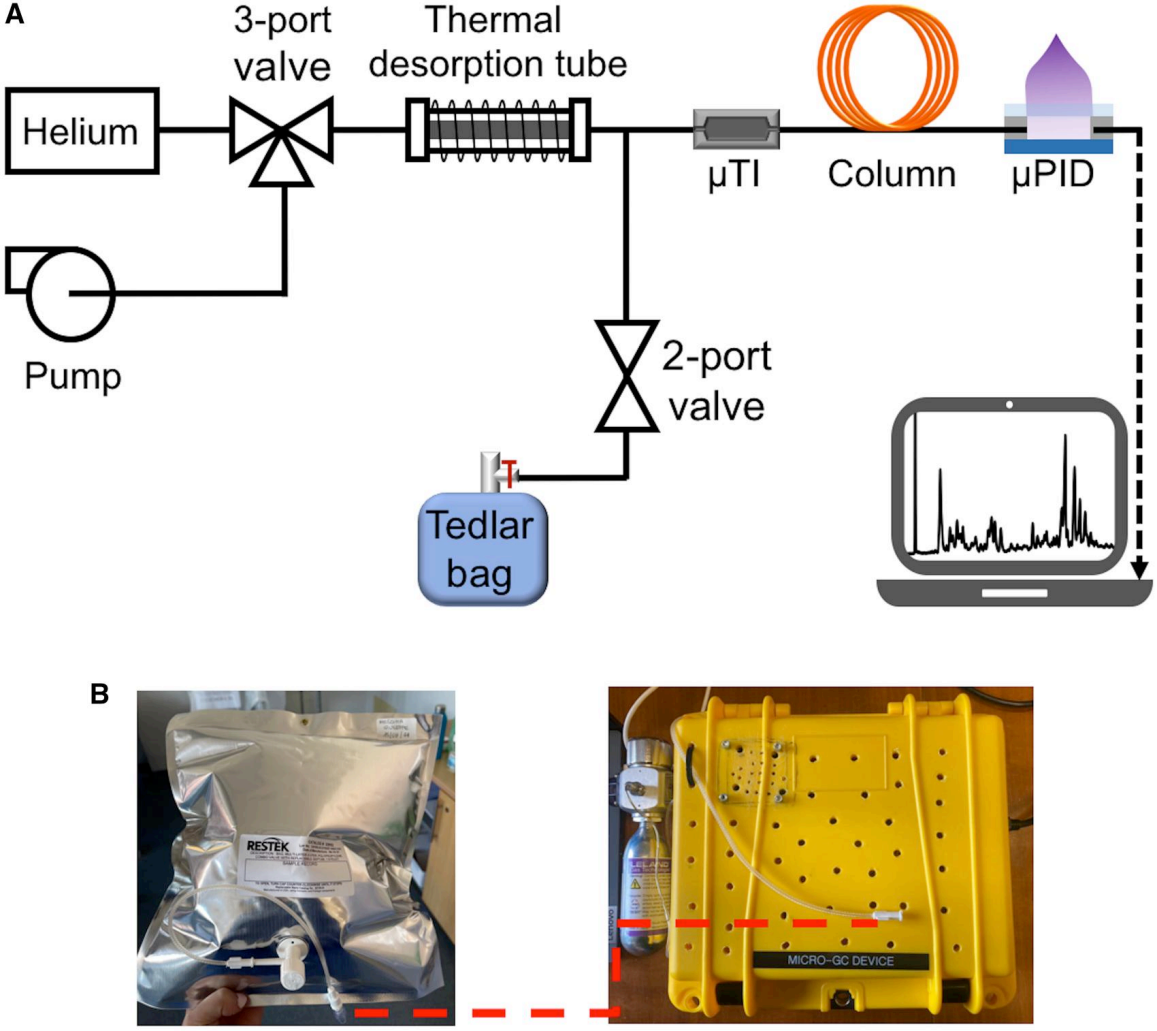
(**A**) Layout of the portable GC device. (**B**) During breath sampling, the subject exhales into a Tedlar bag through a mouthpiece that is connected to a one-way valve. The bag is then connected to the sampling port of the portable GC device.

The study utilized a portable GC system that has been previously described in works published by our study group [7, 8]. The device comprises 2 main modules: a sampling module and an analysing module (Fig. 1). The sampling module includes a sampling tube, thermal desorption tube, valves and a pump. Meanwhile, the analysing module consists of a micro-thermal injector, a 10-m-long column, and a micro-photoionization detector. The entire system is contained in a customized plastic case and weighs <3 kg. This system is illustrated in Fig. 1 for reference.

### Exhaled breath collection and analysis

Patients were instructed to breathe out 1–2 l of air orally into a 5-l Tedlar bag using a one-way mouthpiece and Nafion filter for moisture removal, as shown in Fig. 1B. The process usually takes a few min. The breath analysis was conducted either immediately after the breath sample collection or within 24 h of breath collection. The Tedlar bags were stored under ambient conditions until analysed. During the breath analysis, the Tedlar bag was connected to the sampling port of the portable GC (Fig. 1C). Approximately 350 ml of breath was extracted from the Tedlar bag into the GC for analysis. The operation of the GC was controlled using LabView via a laptop. The total assay time was 30 min, including 5 min of breath sampling time from the Tedlar bag at a flow rate of 70 ml/min (as indicated by the blue path in Fig. 1A), 5 min of desorption/transfer time, 10 min of chromatographic separation time (as indicated by the orange path in Fig. 1A) and 10 min of GC system cleaning time.

### Chromatogram processing and statistical analysis

Continuous variables in the study are presented as mean and standard deviation (SD) and evaluated using the 2-sample *t*-test. Nominal variables are reported as counts and percentages and evaluated using either Pearson’s χ^2^ test or Fisher’s exact test, as appropriate. The statistical analysis was conducted using STATA 14.0 statistical software (StataCorp, 2015, Stata Statistical Software: Release 14; StataCorp LP, College Station, TX). A *P*-value <0.05 was considered statistically significant.

Before conducting breath analysis, chromatogram preprocessing is crucial. This study performs baseline correction, noise reduction, normalization, peak detection, peak area extraction and chromatogram aligning to prepare for subsequent statistical analysis. For a more detailed description of each step, please refer to previous published works [7–11]. A machine learning approach that combines linear discriminant analysis (LDA) and principal component analysis (PCA) was utilized for biomarker selection and statistical analysis [8–12].

## RESULTS

This preliminary analysis included 60 total breath samples, including 30 from NSCLC patients and 30 from healthy controls (HC). A statistically significant gender, age and smoking habit imbalance was observed when the significance level was set to 0.05. The baseline characteristics of recruited patients are summarized in Table 1. Each peak represents one VOC or a set of coeluted ones. Not all peaks may be relevant to NSCLC since some peaks may be from normal metabolic activities, other conditions that a patient may have, or exogenous factors (indoor air background, smoking and use of consumer products, etc.) [13]. Therefore, it is critical to determine which subset of the peaks is most responsible for the differences observed between LC and HC groups. For selecting the optimal subset of peaks (i.e. biomarkers), 40 chromatograms from LC (20) and HC (20) were used as the training set. The remaining 20 chromatograms (10 LC and 10 HC) were used as the testing set.

**Table 1:** Baseline characteristics of patients

Variables	LC	HC	*P*-value
Males	23	12	0.009
Age	67.1 ± 10.0	59.4 ± 12.3	0.005
**Smoke attitude**			0.006
Yes	13	5	
No	7	19	
Ex	10	6	
**Comorbidities**			0.237
Diabetes	3	5	
Hypertension	15	8	
Cardiopathy	6	1	
COVID-19	4	4	
**Stage**			
I	7		
II	6		
III	5		
IV	12		

HC: healthy controls; LC: lung cancer; COVID-19: COronaVIrus Disease of 2019.

Finally, a machine learning approach combining LDA and PCA was used for biomarker selection and statistical analysis [8, 9, 12].

**Figure 2:**
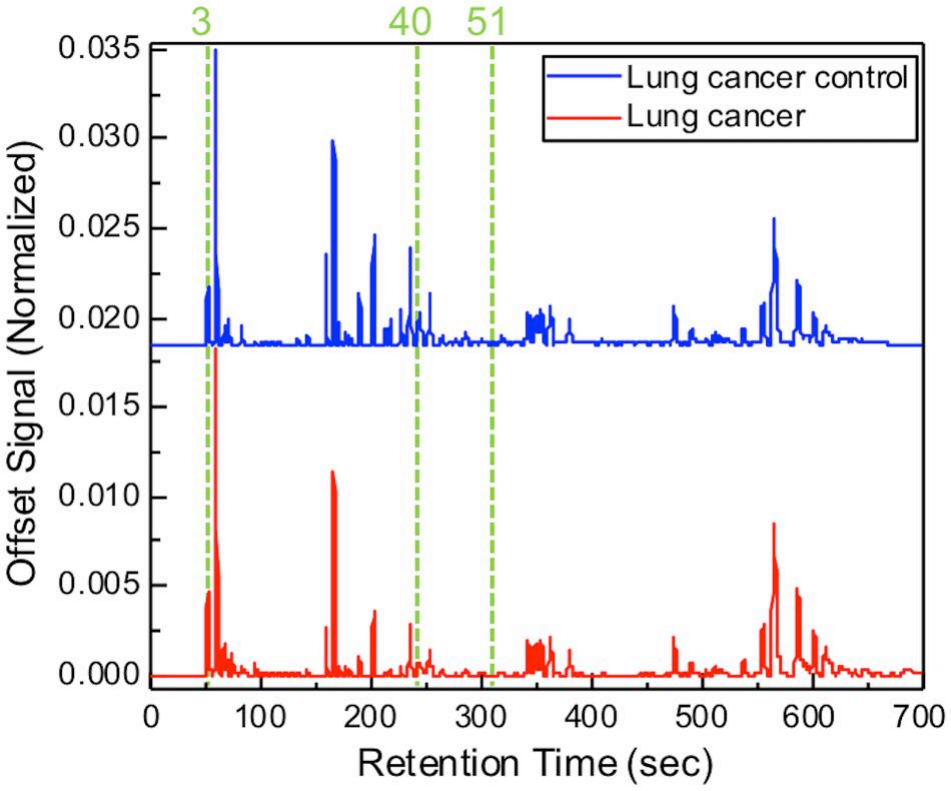
Representative GC chromatogram from a lung cancer and a healthy control patient. The green dashed line and numbers mark the peak positions of the identified biomarkers.

**Figure 3:**
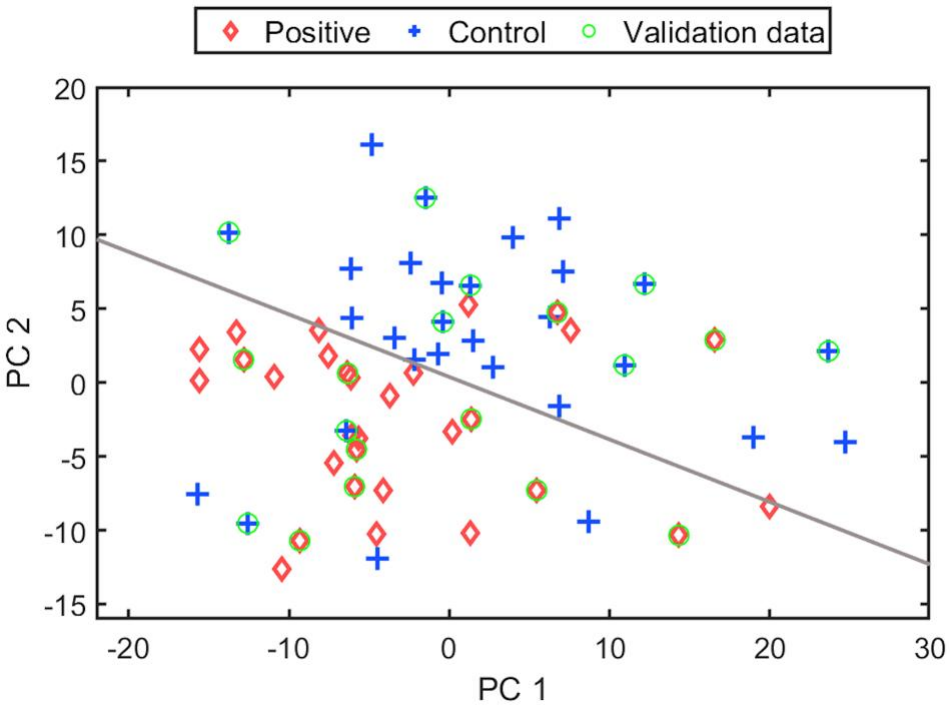
PCA plots to distinguish between lung cancer and healthy control patients using the optimal 3 biomarkers. In the plot, red circles represent lung cancer patients, blue crosses represent healthy control patients, and green circles represent test patients who were not used in the model training. The grey line indicates the model boundary between lung cancer and healthy control patients.

Optimal peak subsets shown in Fig. 2 were identified (mainly from alkane families), which yield the maximum classification accuracy of 85% and the maximum boundary distance to distinguish NSCLC and HC. The main potentially diagnostic VOCs identified were acetone, octane, and 6-methyl 5-heptane 2-one or similar isomers. In addition, a sensitivity of 86.7%, a specificity of 83.3%, a positive predictive value of 83.9%, and a negative predictive value of 85.0% were achieved (Table 2). The corresponding PCA plot is provided in Fig. 3.

**Table 2:** Statistics summary

	Training set			Testing set			Training + testing set		
	LC	HC	Total	LC	HC	Total	LC	HC	Total
Subject number	20	20	40	10	10	20	30	30	60
Positive	18	3	21	8	2	10	26	5	31
Negative	2	17	19	2	8	10	4	25	29
Specificity (%)	85.0			80.0			83.3		
Sensitivity (%)	90.0			80.0			86.7		
Positive predictive value (%)	85.7			80.0			83.9		
Negative predictive value (%)	89.5			80.0			86.2		
Total accuracy (%)	87.5			80.0			85.0		

HC: healthy controls; LC: lung cancer.

## DISCUSSION

The study of VOCs dates back to 1971 when they were first detected in the breath of healthy individuals [14]. In 1985, a team led by Gordon *et al*. [15] developed a breath collection method and used computer-assisted GC/mass spectrometry (MS) to identify various VOCs in the exhaled air of LC patients. Later in 1999, Phillips and colleagues [16] reported over 3400 different VOCs in the exhaled breath of healthy individuals. The findings from these studies suggest a potential correlation between VOCs and the development of LC. Consequently, there has been increased interest in investigating exhaled breath as a non-invasive and potentially valuable means of screening and diagnosing cancer in its early stages. During a breath sample study, researchers typically gather data on both endogenous and exogenous VOCs found in patients. However, monitoring all of these VOCs may not be feasible when trying to detect LC. It is critical to pinpoint a select few metabolic VOCs that are essential for cancer detection. Additionally, determining the number of significant VOCs can help reduce the time and cost associated with collecting data on all VOCs present in breath samples. By concentrating on a select few VOCs, researchers can accurately predict the type of LC present in patients. The carbonyl compounds in exhaled breath play a crucial role in the non-invasive detection of LC; particularly, most of the biomarkers reported previously were alkane, alkane derivatives and benzene derivatives [17–20]. However, a definitive set of breath biomarkers for LC has yet to be established and put into clinical practice. This is primarily due to the many confounding variables that can influence an individual’s breath VOC profile, such as environmental factors, age, gender, diet, medication, smoking history and lifestyle.

Our study population had varying baseline characteristics, but we found that sex and age did not affect the expected pattern of VOCs in exhaled breath, which was consistent with the existing literature. However, a recent review [21] revealed that only a few studies have examined the impact of age and gender on the content of VOCs in exhaled breath, and most of them focused on healthy individuals. For example, Dragonieri *et al.* [22] studied the exhaled breath of 20 healthy individuals and found no significant differences between the 2 age groups (below and above 45 years old). Similarly, Mazzone *et al.* [23] conducted a study on LC prediction using exhaled breath analysis and found that age and gender did not affect the outcomes. They also investigated the impact of tobacco smoking, which is a critical factor to consider when analysing exhaled breath. Some VOCs, such as benzene and acetonitrile, are found in higher concentrations in the breath of smokers than non-smokers. Other VOCs, such as furan, 3-methylfuran, 2,5-dimethylfuran, 2-butanone, octane, and decane, are also present in the exhaled breath of smokers or those exposed to secondhand smoke [24]. Saalberg and Wolff [25] conducted a review article by compiling research from over 52 sources spanning 30 years. They identified 77 possible VOCs that could serve as biomarkers for LC. However, no single substance was deemed sufficient for accurate diagnosis. The most promising breath biomarkers found were 2-butanone and 1-propanol. Instead of relying on a single substance, a combination of substances and their chemometric fingerprint are required for accurate diagnosis.

Schmidt *et al.*’s [26] more recent scoping review analysed 138 publications and 26 methods, presenting 490 potential LC biomarkers. There seems to be no significant difference in the profile of VOCs found in the breath of patients with early or advanced-stage LC [16, 27]. Previous studies have shown limited comparison of VOCs in different types of LC, with some indicating that VOCs are not impacted by the disease [28, 29]. However, some studies have found that patients with adenocarcinoma showed higher levels of 1-butanol and 3-hydroxy-2-butanone [27], while others found that hexane and ethyl benzene levels were elevated in adenocarcinoma compared to squamous cell carcinoma [30]. Additionally, Jia *et al.* [20] found that VOC profiles differed between NSCLC and small cell lung cancer (SCLC), but it was not possible to differentiate between adenocarcinoma and squamous cell carcinomas in NSCLCs. Large cell carcinomas also exhibited a different VOC profile in comparison to other NSCLCs. Therefore, our study focused solely on NSCLC and found that a pattern of VOCs primarily from alkane families can differentiate between NSCLC patients and healthy individuals with 85% accuracy. The main potentially diagnostic VOCs we identified were acetone, octane and 6-methyl 5-heptane 2-one or similar isomers.

The majority of previous studies focusing on LC and using GC were conducted on small samples because of the time needed for collecting and processing the samples. Moreover, the results of all the studies on VOCs are scarcely comparable since there is a large variation in sample size, diverse sample, collection approaches and different analytical and data processing techniques. One of the advantages of using GC-MS systems is their high sensitivity in detecting specific VOCs and measuring their concentrations. However, these systems are expensive and require expertise to operate. Additionally, the breath contents need to be captured and transported to the devices, making them impractical for point-of-care testing. The in-house developed GC device used in this study is portable and cost-effective, eliminating common disadvantages and making it usable for point-of-care analysis. This affordable and portable advanced sensing system offers the significant advantage of enabling testing on larger population samples, with the potential to increase patient diversity. By doing so, it may help in characterizing the distinct pattern of VOCs found in the breath of LC patients. Gathering this information can also be used to improve point-of-care testing devices.

### Limitations

This study has certain limitations that must be taken into account. To begin with, the research was conducted at a single medical centre and the patient sample size was relatively small. Moreover, most patients were elderly and had advanced LC, which could have impacted the results. To have a more comprehensive understanding, larger patient data sets from different clinical locations are necessary, and the current findings must be independently validated through a large-scale, multicentre series, which includes monitoring LC patients over time. Another challenge with breath analysis technologies is the occurrence of false positives and personal variations in VOC profiles, as well as the balance between detection time and specificity. Lastly, to improve the accuracy and stability of VOC selection and achieve better diagnostic performance, additional machine learning tools and feature selection techniques, such as random forest, neural networks, least absolute shrinkage, and selection operator, and minimum redundancy maximum relevance, could be considered as the size of collected breath samples grows.

Further research is necessary to expand the study population, validate the findings on a larger scale, minimize differences between various groups, and eliminate the impact of individual patient characteristics on exhaled VOCs. Additionally, plans will involve including SCLC patients in the study, allowing for the comparison of their VOC pattern with healthy individuals and NSCLC patients. The ultimate goal of this study is to identify a combination of VOCs that have high specificity and sensitivity, which can be used as a global screening fingerprint on a large scale.

## CONCLUSION

Breath analysis through exhalation is a non-invasive method that has promising potential for early cancer screening and diagnosis. However, this technique is not widely used in clinical practice due to a lack of validation. Fortunately, the constant development of various breath analysis techniques has the potential to overcome these challenges. Our study shows preliminary data on the effectiveness of this portable GC technology in analysing exhaled breath VOC signatures. This technology should be further implemented and tested in a larger group of patients, as it holds the potential to become a reliable diagnostic platform for LC with high accuracy.

## Data Availability

The data underlying this article will be shared on reasonable request to the corresponding author.

## Notes

Will be presented as Oral presentation at the 37th EACTS Annual Meeting in Vienna, Austria, 5 October in the Session ‘President’s Choice’.

## ABBREVIATIONS

CT: Computed tomography
GC: Gas chromatography
GCMS: Gas chromatography coupled to mass spectrometry
HC: Healthy controls
LC: Lung cancer
LDA: Linear discriminant analysis
MS: Mass spectrometry
NSCLC: Non-small-cell lung cancer
PCA: Principal components analysis
SCLC: Small cell lung cancer
VOCs: Volatile organic compounds
